# Effects of Gluteal Muscle Strengthening Exercise-Based Core Stabilization Training on Pain and Quality of Life in Patients with Chronic Low Back Pain

**DOI:** 10.3390/medicina60060849

**Published:** 2024-05-23

**Authors:** Seung-Eon Ahn, Mi-Young Lee, Byoung-Hee Lee

**Affiliations:** 1 Graduate School of Physical Therapy, Sahmyook University, Seoul 01795, Republic of Korea; aasn92@hanmail.net; 2 Department of Physical Therapy, Sahmyook University, Seoul 01795, Republic of Korea; mylee@syu.ac.kr

**Keywords:** hip, low back pain, pain, function, strength exercise

## Abstract

*Background*: The World Health Organization reports that back pain is a major cause of disorder worldwide. It is the most common musculoskeletal disorder with limited pain, muscle tension, and stiffness, and 70–80% of all individuals experience it once in their lifetime, with higher prevalence in women than in men. This study aimed to investigate the effects of gluteal muscle strengthening exercise- based core stabilization training (GSE-based CST) on pain, function, fear-avoidance patterns, and quality of life in patients with chronic back pain. *Methods:* This study included 34 patients with non-specific chronic low back pain. Seventeen individuals each were included in GSE-based CST and control groups. The GSE-based CST group performed GSE and CST for 15 min, three times a week for four weeks, and the control group performed CST for 30 min a day, three times a week for four weeks. The numeric pain rating scale was used to evaluate pain before and after treatment, Roland–Morris disability questionnaire was used to evaluate function, fear-avoidance beliefs questionnaire was used to evaluate fear-avoidance patterns, and quality of life was measured using the short form-36. *Results:* In this study, pain, function, and fear-avoidance pattern decreased significantly in both groups (All *p* < 0.05). During the evaluation of quality of life, both groups showed significant increase in physical and mental factors (*p* < 0.05). There were significant differences in pain and quality of life (*p* < 0.05) between the GSE-based CST and control groups. *Conclusions:* Therefore, GSE-based CST can be used as a basis for effective intervention to enhance pain, function, fear-avoidance patterns, and quality of life, emphasizing the need for gluteal muscle strengthening exercises in patients with non-specific chronic back pain in the future.

## 1. Introduction

Low back pain is the most common musculoskeletal disorder and the leading cause of disorder. Between 70–85% of individuals experience low back pain at least once in their lifetime, with the highest prevalence occurring between the ages of 40–69 years. The annual prevalence rate of low back pain is between 15–45%, with a higher prevalence in women than in men [1,2]. Many adults experience low back pain, but 60–90% of them cannot find a specific cause [3]. Low back pain is mostly non-specific and mechanical in nature. Non-specific low back pain often occurs spontaneously, and typically resolves within 4–6 weeks [4]. Specific causes for back pain, such as infections, tumors, osteoporosis, spondyloarthropathies, and trauma, actually represent a minority of such pain syndromes, qualifying for specific therapeutic approaches [5]. Specific causes of back pain are some degenerative conditions, inflammatory conditions, infective and neoplastic causes, metabolic bone disease, referred pain, psychogenic pain, trauma, and congenital disorders [6].

Chronic low back pain is attributed to multiple factors, including delayed or reduced activation of the lumbar multifidus and transversus abdominis muscles during walking or limb movement, and diminished physiological activation of the transversus abdominis. Muscle dysfunction can lead to loss of lumbar support, increasing stress on the surrounding joints and ligaments of the lumbar spine [7]. Chronic low back pain significantly increases the risk of comorbid conditions, including musculoskeletal, neurological, and psychological issues [8]; furthermore, chronic low back pain significantly impacts quality of life, affecting various aspects, including physical and psychological well-being. Individuals with chronic low back pain experience a significant decrease in their quality of life across all dimensions [9,10].

The treatment of patients with low back pain varies, depending on the classification of the patient (specific versus non-specific) and duration of symptoms (acute versus chronic) [11], and typically, exercise therapy targeting the transversus abdominis, lumbar multifidus, and pelvic floor muscles is employed [12]. 

Core stability is the ability to control the position and movement of the trunk over the pelvis, enabling optimal production, transfer, and control of force and motion during integrated movement activities. Core muscle activity is understood as a pre-programmed integration of local single-joint muscles and multi-joint muscles to provide stability and generate movement [13]. The purpose of core stability exercises is to restore normal muscle function to prevent shear forces that can induce segmental stiffness and lumbar spine instability, enhancing pelvic region neuromuscular control, and reducing lumbar injury [14].

Whether hip abductor and external rotator muscle exercises increase the lateral vector force of the patella warrants clarification. These exercises could incorporate hip abductor and external rotator muscle training activities and also induce vastus lateralis muscle activity through muscular cocontraction. Selective gluteus medius muscle activation was induced during the hip abduction and external rotation movements, accompanied by an increase in vastus lateralis muscle activation [15]. 

When considering the influence of muscles that contribute to low back pain, the gluteal muscles play a crucial role as they transmit force from the legs towards the spine during upright activities [16]. Additionally, biomechanically, the gluteal muscles, including the gluteus maximus and medius, play an essential role in stabilizing the trunk and pelvis and transferring force from the legs to the pelvis during all walking activities, while the gluteus maximus stabilizes the pelvis, and the gluteus medius and minimus act as key stabilizers of the pelvis in a single-leg stance position. Therefore, the gluteal muscles play a significant role in transmitting force from the legs towards the spine during upright activities [17]. 

Gluteal examinations and interventions are conducted in individuals with low back pain in clinical practice because the gluteal muscles provide pelvic stability in the coronal and transverse planes and offer a stable foundation for the lumbar spine [18]. Despite the close relationship between low back pain and muscles of the spine and hip joints, studies on concurrent spinal segmental strengthening exercises alongside specific gluteal muscle strengthening exercises in patients with low back pain are lacking. 

The primary outcome measurements of this study are pain, function, and fear avoidance patterns, while the secondary outcome measurement is quality of life. Low back pain manifests as pain localized below the costal margin and above the gluteal fold, along with muscle tension or stiffness, and its most significant symptoms are pain and disorder [19]. Low back pain is the most common cause of functional disability, and functional disability assessment is one of the most important components of medical services [20]. Individuals with chronic low back pain often exhibit avoidance beliefs towards movement, which manifest as fear of movement [21]. Negative beliefs about pain lead to negative information about the illness, causing patients to imagine the worst possible outcomes and creating fear that leads to avoidance of movement [22]. Additionally, quality of life is a multidimensional concept used to measure an individual’s health. Over the past few years, there has been significant interest in investigating the impact of physical and psychological issues on overall quality of life, as the shift from biomedical issues to biopsychosocial issues has occurred. Furthermore, the transition to psychosocial issues has been shown to play a crucial role in ensuring positive outcomes from both the clinical and patient perspectives. Therefore, based on the objectives of this study, the following hypotheses were formulated:

**Hypothesis** **1.** *Gluteal muscle strengthening exercise-based core stabilization training (GSE-based CST) will have differential effects on pain in patients with chronic low back pain.*

**Hypothesis** **2.** *GSE-based CST will have differential effects on function in patients with chronic low back pain.*

**Hypothesis** **3.** *GSE-based CST will have differential effects on fear avoidance patterns in patients with chronic low back pain.*

**Hypothesis** **4.** *GSE-based CST will have differential effects on quality of life in patients with chronic low back pain.*

**Hypothesis** **5.** *There will be differential effects on pain, function, fear avoidance patterns, and quality of life between GSE-based CST and the control group.*

## 2. Materials and Methods

### 2.1. Participants

This study included 34 adult patients with chronic low back pain at the S Hospital, Seoul, South Korea. Before recruiting the participants for this study, we performed a power analysis using G*Power version 3.1.9.7 20 (Heinrich-Heine Universität, Düsseldorf, Germany); an overall effect size index of 0.85 was obtained for all the outcome measures, with a probability of 0.05, to minimize type II errors (power of 80%). Because the estimated target sample size was 36, we recruited 40 participants who underwent physical therapy. 

Inclusion criteria were individuals aged ≥20 years who experienced daily low back pain for a minimum of eight weeks, with pain localized just below the iliac crest to the gluteal fold, and with or without leg pain. This study targeted individuals experiencing pain or discomfort persisting for ≥12 weeks. Exclusion criteria included individuals with red-flag conditions, such as cancer, metabolic disorders, rheumatoid arthritis, osteoporosis, or long-term steroid use; those with acute pain [23]; those with immediate issues due to nerve compression; those who underwent lumbar surgery; pregnant women [24]; those with fractures, gastrointestinal or bladder dysfunction, or central nervous system problems; and individuals with sensory deficits or numbness in the lower extremities [25].

All the participants signed a consent form after the procedure, and the purpose of the study was explained. This study was approved by the Sahmyook University Institutional Review Board (approval number: SYU 2022-07-023) and Clinical Research Information Service (KCT0007883). The participants fully understood the objectives and procedures used in the study. The study adhered to the ethical principles of the Declaration of Helsinki.

### 2.2. Experimental Procedure

This study obtained approval for review of medical records confirming clinical characteristics, such as onset date, cause of onset, and medical history before the study. General characteristics were assessed based on age, height, weight, and body mass index (BMI). Physical therapists responsible for examination conducted pre-tests one hour before group allocation. To minimize bias and errors in the experiment, the researchers used the Research Randomizer program (http://www.randomizer.org/, accessed on 4 September 2022) to randomly assign the participants to the two groups, thereby minimizing bias and ensuring a fair experiment. Among the 40 recruited participants, three were excluded because they did not meet the selection criteria of a numeric pain rating scale (NPRS) score of ≤2, and three were excluded because they did not meet the age criteria. Consequently, the final allocation resulted in 17 participants each in the GSE-based CST and control groups based on the treatment method. The GSE-based CST group underwent gluteal strengthening exercises for 15 min and CST for 15 min three times a week for four weeks, while the control group underwent CST for 30 min three times a week for four weeks. Pre- and post-evaluation items included a NPRS, disability index, fear avoidance behavior, and quality of life measurements. Pre- and post-evaluations were conducted independently and at the same examination location to ensure consistency, and the therapists responsible for the evaluations did not administer any physical therapy intervention. 

The physical therapists involved in the intervention had at least three years of experience and were well-versed in potential issues that may arise when applying GSE-based CST and CST (control group). The same therapist administered all treatments to ensure consistency. 

### 2.3. Training Program

In this study, GSE-based CST was conducted to improve pain, function, fear avoidance behavior, and quality of life in patients with low back pain.

#### 2.3.1. GSE-Based CST

Gluteal muscle strengthening exercise-based CST involves performing gluteal strengthening exercises and promoting spinal stability in patients with nonspecific chronic low back pain. This approach aims to strengthen the core muscles by targeting the gluteal muscles. The gluteal strengthening exercise-based CST program presented in this study is based on the exercises presented in Table 1 [18,26,27,28]. The exercise program was reorganized, and the CST program was based on the exercises presented in Table 1 [27,28].

**Table 1:** Gluteal muscle strengthening exercise-based core stabilization training (GSE-based CST).

- Gluteal exercises: clamshell exercise, donkey kick exercise, single-leg bridge exercise, adductor stretch exercise, and adductor muscles abduction + bridge exercise - Core stabilization training: abdominal muscle exercises, dead bug exercise, and crunch exercise (Table 2) - Section: 15 times, 3 sets each																			
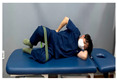				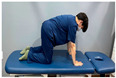				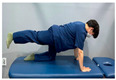				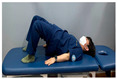				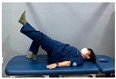			
Clamshell exercise				Donkey kick exercise								Single-leg bridge exercise							
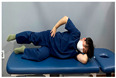					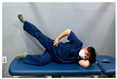					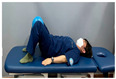					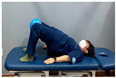				
Adductor stretch exercise										Adductor muscles abduction + bridge exercise									
Total exercise time is 30 min, with rest periods between sets lasting 30–40 s.																			

**Table 2:** Core stabilization training (CST).

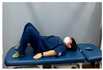	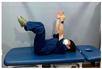	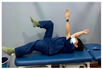	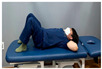	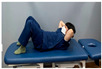
Abdominal muscle exercises	Dead bug exercise		Crunch exercise	
Total exercise time is 30 min, with rest periods between sets lasting 30–40 s.				

#### 2.3.2. CST

For CST, the participant lies on a table, bends the knee and hip joints, activates the abdominal muscles, and holds the contraction without changing the muscle length for 10 s, lying on the table and activating the abdominal muscles while crossing the opposite arm and leg and moving, lying on the table and bending the knee and hip joints, and contracting the upper abdominal muscles by contracting the upper body until contraction of the upper abdominal muscles is felt [27,28]. This movement was repeated 15 times in three sets. The intensity of the training was set at 70% of the maximum strength that the participant could exert in one repetition. Each exercise was performed in three sets, with 15 repetitions for each set, and was conducted three times a week for four weeks (Table 2).

#### 2.3.3. Outcome Measures

Pain was assessed using the NPRS. The NPRS is used to assess chronic low back pain. It ranges from 0 cm, representing no pain, to 10 cm, representing severe pain. The NPRS is a simple and highly reproducible tool for patients to express pain intensity. It has demonstrated high sensitivity and reliability, with an intraclass correlation coefficient (ICC) of 0.96 [29]. To evaluate patient status, independent locations were provided in the same place to ensure that the patients could focus on the evaluation, and pre- and post-evaluations were conducted.

Functional status was measured using the Roland–Morris disability questionnaire (RMDQ). The RMDQ is a health status measurement tool used to assess physical disability in patients with low back pain. It is a questionnaire-based tool suitable for assessing short-term changes in low back pain. The score is calculated by summing the number of items checked by the respondents. There are 24 items, and the respondents checked the items that applied to their condition. The score ranges from 0 (no disability) to 24 (maximum disability), with a higher score indicating greater disability. It is typically administered to patients with mild-to-moderate low back pain. The test-retest reliability of the 24 items indicates an ICC ranging between 0.42–0.91 [30]. This study conducted pre- and post-evaluations at independent locations in the same area to ensure that patients could focus on the evaluation.

Fear avoidance behavior was measured using the fear avoidance belief questionnaire (FABQ). The FABQ was developed to assess fear avoidance behavior in patients with low back pain. The FABQ is divided into two subscales: fear avoidance beliefs about physical activity (FABQ-PA) and fear avoidance beliefs about work (FABQ-W). The FABQ-PA consists of items related to physical activity, numbered from 1 to 5. The FABQ-W consists of items related to work, numbered from 6 to 16. Each item was scored from 0 to 6 points. Items 2–5 had a maximum score of 24 points each, whereas items 6, 7, 9, 10, 11, 12, and 15 had a maximum score of 42 points each. A higher total score indicates greater fear avoidance beliefs. The test-retest reliability of the FABQ was high (ICC = 0.9), which correlated with the RMDQ. The correlation coefficients for the FABQ, FABQ-W subscale, and FABQ-PA subscale were 0.52, 0.63, and 0.51, respectively [31]. This study conducted pre- and post-evaluations at independent locations in the same location to ensure that patients focus on the evaluation. 

The short form-36 (SF-36), a general health-related quality of life measurement tool, was used to assess quality of life. The SF-36 measures general health status, but can also assess health status in specific disease populations. The SF-36 consists of physical (physical component summary, PCS) and mental (Mental Component Summary, MCS) components. The scores of the scales were transformed to obtain values between 0–100, with higher scores indicating better health status. A combination of physical and mental components is referred to as overall health (global health, GH). Higher scores indicate a higher quality of life in the respective domains. The test-retest reliability of the Korean version of the SF-36 ranges between 0.710–0.895, and the internal consistency, measured by Cronbach’s alpha, ranges between 0.930–0.938 [32]. This study conducted pre- and post-evaluations at independent locations in the same area to ensure that patients focus on the evaluation.

#### 2.3.4. Data Analysis

All statistical analyses were performed using SPSS ver. 23.00 software (SPSS Inc., Chicago, IL, USA). Descriptive statistics for the baseline variables were calculated to characterize and compare the two study groups. The Shapiro–Wilk test was used to determine the distribution of the general properties and outcome measures of the subjects, and means with standard deviations were reported for normally distributed variables. A paired *t*-test was used to compare the dependent variables before and after the intervention within each group (intragroup). Independent *t*-tests were performed to compare the differences between groups. Statistical significance was set at a *p*-value of <0.05. There was a correlation between the Morris assessment and fear avoidance, thus these two independent variables are not independent. Therefore, the significance level for RMDQ and FABQ in this study was set at 0.025 two-tailed.

## 3. Results

### 3.1. General Characteristics of Participants

In this study, the participants consisted of 34 patients with chronic low back pain divided into two groups: the GSE-based CST Group with 17 participants, and the control group with 17 participants. The homogeneity test results indicated homogeneity between the groups, as shown in Table 3.

### 3.2. Comparison of NPRS, RMDQ, and FABQ

Before the experiment, there were no significant differences between the two groups in the pre-test values of the NPRS, RMDQ, and FABQ, confirming homogeneity between the groups. In the comparison between pre- and post-treatments within each group, the NPRS significantly decreased from 6.76 to 2.41 cm (*p* < 0.05) in the GSE-based CST group, and from 6.00 to 2.94 cm (*p* < 0.05) in the control group. For RMDQ, there was a significant reduction from 7.00 to 2.47 points in the GSE-based CST group (*p* < 0.05), and from 6.88 to 3.41 points in the control group (*p* < 0.05), indicating a statistically significant decrease after treatment. For FABQ, there was a significant reduction from 55.11 to 32.47 points in the GSE-based CST group (*p* < 0.05), and from 54.41 to 36.29 points in the control group (*p* < 0.05), indicating a statistically significant decrease after the treatment. 

When examining the difference in NPRS between the groups, the difference in pre- and post-treatment values were 4.35 and 3.05 cm for the GSE-based CST and control groups, respectively. This indicates a statistically significant difference between the GSE-based CST and control groups (*p* < 0.05). When examining the differences in RMDQ and FABQ between the groups, the GSE-based CST group showed a greater amount of change than the control group. However, there was no significant statistical difference between the two groups (Table 4). 

### 3.3. Comparison of QOL

Before the experiment, there was no significant difference in the pre-test values of the SF-36 PCS, MCS, and GH subscales between the two groups, indicating that the groups were homogeneous. In the comparison within each group before and after the experiment, the PCS scores significantly increased in both the GSE-based CST and control groups. Specifically, in the GSE-based CST group, the PCS score increased from 39.77 to 77.35 (*p* < 0.05), and in the control group, it increased from 49.81 to 70.66 (*p* < 0.05). 

In both the GSE-based CST and control groups, the MCS scores significantly increased after the treatment. Specifically, in the GSE-based CST group, the MCS score increased from 47.86 to 79.01 (*p* < 0.05), whereas in the control group, it increased from 54.28 to 70.87 (*p* < 0.05). In both the GSE-based CST and control groups, the GH scores significantly increased after the treatment. Specifically, in the GSE-based CST group, the GH score increased from 43.82 to 78.18 (*p* < 0.05), and in the control group, it increased from 52.04 to 70.76 (*p* < 0.05). When comparing the differences in PCS between the two groups, the pre-post difference values were 37.57 points for the GSE-based CST group and 20.84 points for the control group. The increase in PCS was statistically significant in the GSE-based CST group compared with that in the control group (*p* < 0.05). 

The difference in MCS between the pre- and post-measurements was 31.15 points in the GSE-based CST group and 16.59 points in the control group. There was a significant statistical difference between the two groups (*p* < 0.05). Global health showed a difference of 34.36 points for the GSE-based CST group and 18.72 points for the control group between the measurements before and after the treatment, with a statistically significant difference between the two groups (*p* < 0.05) (Table 5).

## 4. Discussion

### 4.1. Changes in Pain

Pain is defined as actual or potential tissue damage associated with unpleasant sensations and emotional experiences [2]. Low back pain refers to pain localized below the lower rib margin and above the gluteal fold, and is often accompanied by muscle tension or stiffness. The most significant symptoms of non-specific low back pain are pain and disability [19]. The characteristics of chronic low back pain include pain or discomfort persisting for 7–12 weeks or longer, with or without symptoms radiating to the legs [13]. Furthermore, regarding the influence of muscles on low back pain, hip muscles play a crucial role in transmitting forces from the legs to the spine during upright activities, theoretically affecting the development of low back pain [16]. 

Within-group differences in this study show that the GSE-based CST group showed a statically significant decrease in NPRS from pre- to post-treatment, and the control group also showed a statistically significant decrease in NPRS from pre- to post-treatment. These findings indicate that Hypothesis 1 was accepted. And regarding between-group differences, only the GSE-based CST group showed a statistically significant decrease in NPRS compared to the control group, thus supporting Hypothesis 5.

Fukuda et al. [18] conducted a study on 70 patients with non-specific low back pain, investigating the effects of exercise therapy, CST, and gluteal muscle strengthening exercises. According to the study results, there was a significant decrease in visual analogue scale (VAS) score from 5.5 to 2.3 points in an experimental group (*p* < 0.05). Bade et al. [27] conducted a study on 90 patients with non-specific low back pain, implementing back pain-related training and hip-strengthening exercises to investigate their effects on the VAS. They observed a decrease in VAS scores from 5.1 to 1.1 in an experimental group and from 5.4 to 1.9 in a control group, showing a significant difference between the two groups (*p* < 0.05). Similar to previous studies, our study confirmed significant differences within the groups in the evaluation of the NPRS.

Chronic low back pain often manifests as localized pain and a considerable proportion of widespread pain, which may indicate a worse prognosis. However, exercise therapy can help reduce pain and improve or maintain function in patients with chronic low back pain [6]. Furthermore, evidence suggests that exercise modulation training in individuals with recurrent low back pain reduces pain intensity [33]. In individuals with non-specific chronic low back pain, core stabilization exercises have been shown to be more effective in reducing pain and improving functional status compared to traditional exercises [34]. This study demonstrated that exercise-based CST, which focused on strengthening the muscles around the spine, improved the tension in the muscle fibers associated with chronic pain. Additionally, it increased the activity of the surrounding muscles and enhanced the role of joint mobility, leading to changes in the muscle activity of the buttocks and spine and reducing excessive tension around the waist and pelvis, thereby improving pain.

### 4.2. Changes in Function

Back pain is the most common cause of functional disability, and functional disability assessment is one of the most important components of medical services [20]; patients with chronic pain recover function, pain decreases, and function improves [35]. In relation to chronic pain, functional disability has been shown to have a negative impact on patients’ quality of life, encompassing not only physical but also psychological and social aspects [21]. In this study, within-group differences in this study show that the GSE-based CST group experienced statistically significant increases in RMDQ from pre- to post-treatment, and the control group also experienced statistically significant increases in RMDQ from pre- to post-treatment. These findings indicate that Hypothesis 2 was accepted. Regarding between-group differences, although the GSE-based CST group exhibited more changes than the control group, no significant differences were observed between the groups, and thus Hypothesis 5 was rejected.

Bade et al. [27] compared the effects of hip exercises in a study involving 30 patients with non-specific chronic or recurrent low back pain. In the study, experimental groups 1, 2, and 3 underwent hip rotation stretching, multidirectional hip stretching, and hip strengthening training, respectively. The results showed a significant difference among the three groups, with experimental group 1 decreasing from 18.2 to 14.8 points, 18.6 to 13.2 points in experimental group 2, and 18.6 to 9.6 points in experimental group 3 (*p* < 0.05). Chronic low back pain is a significant health issue, with the most important symptoms being pain and functional impairment [30]. Patients with disabilities caused by recurrent back pain may experience limitations in daily activities and inappropriate neuromuscular adaptations to maintain function [36]. 

In the treatment of chronic back pain, programs vary widely, but stabilization exercises are considered most effective, and CST has been shown to reduce pain and improve functional disability [37]. Core stabilization training based on gluteal muscle-strengthening exercises is believed to enhance the stability in the spine and pelvis, leading to reduced back pain and improved function. Although no significant differences were observed between the groups, this could be attributed to the influence of the intervention duration, as suggested by previous studies. Therefore, differences between the groups may emerge in functional aspects if the experiment was evaluated and conducted over a longer period. Furthermore, in this study, the gluteal muscle strengthening exercise-based CST showed an overall improvement in lumbar function, which was considered to be effectively enhanced.

### 4.3. Changes in Fear Avoidance Behavior

Individuals with chronic low back pain often exhibit fear avoidance beliefs regarding movement, which manifests as kinesiophobia [21]. Negative beliefs about pain can lead to physical, psychological, and quality of life problems. These negative beliefs can instill fear in patients, leading them to imagine the worst possible outcomes related to their condition and avoid movement. Such outcomes can have negative repercussions, leading to actual or anticipated painful experiences and exacerbating the detrimental effects. Within the initial six months, patients experiencing fear of movement often reported more pain and disability. This study used the FABQ. Within-group differences in this study show that the GSE-based CST group experienced statistically significant increases in FABQ from pre- to post-treatment, and the control group also experienced statistically significant increases in FABQ from pre- to post-treatment. These findings indicate that Hypothesis 3 was accepted. Regarding between-group differences, although the GSE-based CST group exhibited more changes than the control group, no significant differences were observed between the groups, and thus Hypothesis 5 was rejected.

Kim et al. [38] performed simulated horseback riding exercises in 48 patients with chronic low back pain. The FABQ scores for the physical component decreased from 15.35 to 8.80 in an experimental group, while in a control group, they decreased from 11.93 to 11.10. There was a significant within-group difference in the experimental group (*p* < 0.05) but not in the control group. Back pain is very common; however, when back pain disorders persist beyond the normal healing time and progress to chronicity, a specific underlying mechanism cannot be assumed. 

Chronic back pain is generally accepted to be multifactorial in nature, with psychological and social factors as characteristic elements that can influence pain-avoidance behaviors that may impact disability, which varies individually [39]. Psychological factors influence the onset of chronic back pain and can contribute to fear avoidance patterns. These patterns have been identified as important psychosocial variables related to back pain in patients with chronic disabilities [40], and reductions in fear avoidance patterns have been shown to correlate with decreases in pain and disability in chronic back pain [41]. Pain and functional disability are influenced not only by pathological issues but also by psychosocial factors. In this study, pain reduction and functional improvement were observed in the GSE-based CST group. According to these results, significant differences were also observed in fear avoidance patterns in the GSE-based CST group due to pain reduction and functional improvement. 

### 4.4. Change in Quality of Life

The concept of determinants of health-related quality of life has evolved since the 1980s to encompass broader aspects of quality of life that can clearly impact physical or mental health [42]. The SF-36 is a highly popular tool for evaluating health-related quality of life, and it broadly consists of physical and mental components that can be summarized into two factors [43,44]. This study used the SF-36. Within-group differences indicate that both the GSE-based CST group and the control group experienced statistically significant increases in PCS, MCS, and GH from pre- to post-treatment. This suggests that Hypothesis Fourth was accepted. Regarding between-group differences, the GSE-based CST group showed statistically significant increases in PCS, MCS, and GH compared to the control group, supporting Hypothesis Fifth for PCS, MCS, and GH.

In a previous study [45], two experimental groups of 66 patients with non-specific low back pain (experimental group 1, which performed hip stretching exercises alongside core stabilization exercises, and experimental group 2, which performed gluteal strengthening exercises alongside core stabilization exercises)conducted a comparative study in a control group performing core stabilization exercises with concurrent skin contact and those performing stabilization exercises alongside manual therapy or solely performing stabilization exercises. According to SF-36 scores for the PCS, the experimental group 1 increased from 29.69 to 47.51 points, experimental group 2 increased from 30.42 to 47.34 points, and the control group increased from 30.44 to 38.99 points, showing significant differences among the three groups (*p* < 0.05). According to results from MCS, experimental group 1 increased from 46.46 to 60.91 points, experimental group 2 increased from 46.69 to 59.56 points, and the control group increased from 46.26 to 54.29 points, indicating significant differences among the three groups (*p* < 0.05). 

Quality of life is significantly correlated with pain acceptance and participation in pain-related activities. Lower levels of pain are associated with better quality of life, and pain plays a crucial role in explaining aspects of quality of life in physical and social domains [46]. Patients who experience pain often have a significantly lower quality of life compared to those who do not experience pain. Patients with back pain or multiple pain sites tend to experience pain and disorder, which negatively impacts their overall health and consequently lowers their quality of life. Changes in pain show the strongest correlation with quality of life [47]. Health-related quality of life is influenced by chronic pain in various domains, such as physical and mental health, social relationships, and functional abilities [48]. 

Quality of life is associated with pain and functioning. As pain decreases and function improves, individuals experience higher levels of achievement and satisfaction with their daily activities. GSE-based CST resulted in decreased back pain, improved lumbar function, and reduced fear of pain. Improved factors are believed to increase the overall satisfaction with life for patients with back pain, affecting both physical and mental aspects, resulting in overall improvement. The GSE-based CST group showed significant differences compared to the control group. Considering these results, it seems that supplementing CST with gluteal strengthening exercises, as seen in the GSE-based CST, is more effective than solely performing the traditional CST (control group) and could be an effective intervention for enhancing the quality of life in patients with chronic back pain.

Our study has limitations. First, the selection of participants was limited to patients receiving treatment at the current hospital, which may restrict the generalizability of the findings to a broader population. Second, the small sample size of 34 participants is small. This may have limited the statistical power and generalizability of the study findings. As a result, it may be difficult to generalize the findings of the study to all patients with back pain, given the limited sample size and restriction to patients receiving treatment at a specific hospital. Furthermore, although short-term effects could be observed after 12 sessions of treatment administered three times a week for four weeks, it was challenging to demonstrate long-term effects of applying exercise interventions for more than four weeks.

Moreover, SES-based CST should be applied to a larger number of patients and implemented in long-term treatment plans to achieve intervention efficacy. Future studies should quantify and refine gluteal strengthening exercise-based CST for application in clinical settings, and follow-up studies should be conducted to evaluate the effects of interventions based on gluteal muscle strengthening exercises on functional and fear avoidance pattern assessments. 

## 5. Conclusions

This study aimed to investigate the effects of GSE-based CST on pain, function, fear avoidance patterns, and quality of life in patients with non-specific low back pain. In this small randomized clinical trial of patients with chronic low back pain, a 4-week exercise intervention program of gluteal strengthening and core stabilization and a 4-week core stabilization program were both associated with significantly reduced pain, disability, fear avoidance, and improved quality of life. However, the combined gluteal strengthening and core stabilization group made significantly greater gains than the core stabilization group. Based on this study, it can be proposed that gluteal muscle strengthening exercise-based core stabilization training is an effective intervention method for future clinical practice.

## Figures and Tables

**Table 3:** General characteristics of participants (N = 34).

Characteristics	GSE-Based CST Group (n = 17)	Control Group (n = 17)	*t* (*p*)
Age (years)	44.52 (11.50) ^a^	46.70 (10.99)	0.564 (0.577)
Height (cm)	167.29 (7.53)	165.41 (7.85)	−0.713 (0.481)
Weight (kg)	60.82 (10.44)	59.35 (10.01)	−0.419 (0.678)
BMI (kg/m^2^)	21.59 (2.16)	21.62 (2.82)	0.037 (0.971)

^a^ M(SD), mean (standard deviation); BMI = body mass index; GSE-based CST = gluteal muscle strengthening exercise-based core stabilization training; control group = core stabilization training (CST).

**Table 4:** Comparison of NPRS, RMDQ, and FABQ.

Parameters		GSE-Based CST Group (n = 17)	Control Group (n = 17)	*t* (*p*)
NPRS (cm)	Before	6.76 (1.71) ^a^	6.00 (1.76)	−1.280 (0.210)
	After	2.41 (1.12)	2.94 (1.59)	
	Before-after	4.35 (1.36)	3.05 (1.47)	−2.651 (0.012)
	*t* (*p*)	13.133 (<0.001)	8.534 (<0.001)	
RMDQ (scores)	Before	7.00 (3.77)	6.88 (4.04)	−0.088 (0.931)
	After	2.47 (2.00)	3.41 (3.14)	
	Before-after	4.52 (2.89)	3.47 (1.94)	−1.252 (0.220)
	*t* (*p*)	6.448 (<0.001)	7.375 (<0.001)	
FABQ (scores)	Before	55.11 (19.10)	54.41 (16.55)	−0.115 (0.909)
	After	32.47 (16.34)	36.29 (19.68)	
	Before-after	22.64 (16.31)	18.11 (11.93)	−0.924 (0.362)
	*t* (*p*)	5.723 (<0.001)	6.261 (<0.001)	

^a^ Mean (Standard Deviation); GSE-based CST = gluteal muscle strengthening exercise-based core stabilization training; control group = core stabilization training (CST); NPRS = numeric pain rating scale; RMDQ = Roland–Morris disability questionnaire; FABQ = fear avoidance beliefs questionnaire.

**Table 5:** Comparison of QOL.

Parameters		GSE-Based CST Group (n = 17)	Control Group (n = 17)	*t* (*p*)
PCS (scores)	Before	39.77 (21.78) ^a^	49.81 (20.54)	1.382 (0.177)
	After	77.35 (12.73)	70.66 (18.90)	
	Before-after	−37.57 (20.42)	−20.84 (13.68)	−2.805 (0.009)
	*t* (*p*)	−7.584 (<0.001)	−6.283 (<0.001)	
MCS (scores)	Before	47.86 (22.78)	54.28 (17.87)	0.914 (0.367)
	After	79.01 (14.18)	70.87 (16.75)	
	Before-after	−31.15 (23.47)	−16.59 (15.23)	−2.146 (0.040)
	*t* (*p*)	−5.473 (<0.001)	−4.492 (<0.001)	
GH (scores)	Before	43.82 (20.61)	52.04 (17.85)	1.244 (0.223)
	After	78.18 (11.64)	70.76 (17.19)	
	Before-after	−34.36 (20.66)	−18.72 (11.56)	−2.724 (0.012)
	*t* (*p*)	−6.857 (<0.001)	−6.676 (<0.001)	

^a^ Mean (Standard Deviation); GSE-based CST = gluteal muscle strengthening exercise-based core stabilization training; control group = core stabilization training (CST); PCS = physical component summary; MCS = mental component summary; GH = global health.

## Data Availability

Data are contained within the article.
